# Supplementary material for: Iterative improvement in the automatic modular design of robot swarms
Source: PeerJ Comput Sci. 2020 Dec 7;6:e322. doi: 10.7717/peerj-cs.322 (PMC7924708; doi:10.7717/peerj-cs.322)
Supplement: Supplemental Information 3 [file peerj-cs-06-322-s003.zip › argos3/doc/api/standalone/a00566_source.html]

ARGoS: plugins/simulator/entities/cylinder\_entity.cpp Source File


- Main Page
- Related Pages
- Namespaces
- Classes
- Files

- File List
- File Members

# plugins/simulator/entities/cylinder\_entity.cpp

Go to the documentation of this file.

```
00001 
00007 #include "cylinder_entity.h"
00008 #include <argos3/core/utility/math/matrix/rotationmatrix3.h>
00009 #include <argos3/core/simulator/space/space.h>
00010 #include <argos3/core/simulator/simulator.h>
00011 #include <argos3/plugins/simulator/media/led_medium.h>
00012 
00013 namespace argos {
00014 
00015    /****************************************/
00016    /****************************************/
00017 
00018    CCylinderEntity::CCylinderEntity() :
00019       CComposableEntity(NULL),
00020       m_pcEmbodiedEntity(NULL),
00021       m_pcLEDEquippedEntity(NULL),
00022       m_fMass(1.0f),
00023       m_pcLEDMedium(NULL) {
00024    }
00025 
00026    /****************************************/
00027    /****************************************/
00028 
00029    CCylinderEntity::CCylinderEntity(const std::string& str_id,
00030                                     const CVector3& c_position,
00031                                     const CQuaternion& c_orientation,
00032                                     bool b_movable,
00033                                     Real f_radius,
00034                                     Real f_height,
00035                                     Real f_mass) :
00036       CComposableEntity(NULL, str_id),
00037       m_pcEmbodiedEntity(
00038          new CEmbodiedEntity(this,
00039                              "body_0",
00040                              c_position,
00041                              c_orientation,
00042                              b_movable)),
00043       m_pcLEDEquippedEntity(
00044          new CLEDEquippedEntity(this, "leds_0")),
00045       m_fRadius(f_radius),
00046       m_fHeight(f_height),
00047       m_fMass(f_mass) {
00048       AddComponent(*m_pcEmbodiedEntity);
00049       AddComponent(*m_pcLEDEquippedEntity);
00050    }
00051 
00052    /****************************************/
00053    /****************************************/
00054 
00055    void CCylinderEntity::Init(TConfigurationNode& t_tree) {
00056       try {
00057          /* Init parent */
00058          CComposableEntity::Init(t_tree);
00059          /* Parse XML to get the radius */
00060          GetNodeAttribute(t_tree, "radius", m_fRadius);
00061          /* Parse XML to get the height */
00062          GetNodeAttribute(t_tree, "height", m_fHeight);
00063          /* Parse XML to get the movable attribute */
00064          bool bMovable;
00065          GetNodeAttribute(t_tree, "movable", bMovable);
00066          if(bMovable) {
00067             /* Parse XML to get the mass */
00068             GetNodeAttribute(t_tree, "mass", m_fMass);
00069          }
00070          else {
00071             m_fMass = 0.0f;
00072          }
00073          /* Create embodied entity using parsed data */
00074          m_pcEmbodiedEntity = new CEmbodiedEntity(this);
00075          AddComponent(*m_pcEmbodiedEntity);
00076          m_pcEmbodiedEntity->Init(GetNode(t_tree, "body"));
00077          m_pcEmbodiedEntity->SetMovable(bMovable);
00078          /* Init LED equipped entity component */
00079          m_pcLEDEquippedEntity = new CLEDEquippedEntity(this);
00080          AddComponent(*m_pcLEDEquippedEntity);
00081          if(NodeExists(t_tree, "leds")) {
00082             /* Create LED equipped entity
00083              * NOTE: the LEDs are not added to the medium yet
00084              */
00085             m_pcLEDEquippedEntity->Init(GetNode(t_tree, "leds"));
00086             /* Add the LEDs to the medium */
00087             std::string strMedium;
00088             GetNodeAttribute(GetNode(t_tree, "leds"), "medium", strMedium);
00089             m_pcLEDMedium = &CSimulator::GetInstance().GetMedium<CLEDMedium>(strMedium);
00090             m_pcLEDEquippedEntity->AddToMedium(*m_pcLEDMedium);
00091          }
00092          UpdateComponents();
00093       }
00094       catch(CARGoSException& ex) {
00095          THROW_ARGOSEXCEPTION_NESTED("Failed to initialize the cylinder entity.", ex);
00096       }
00097    }
00098 
00099    /****************************************/
00100    /****************************************/
00101 
00102    void CCylinderEntity::Reset() {
00103       /* Reset all components */
00104       m_pcEmbodiedEntity->Reset();
00105       m_pcLEDEquippedEntity->Reset();
00106       /* Update components */
00107       UpdateComponents();
00108    }
00109 
00110    /****************************************/
00111    /****************************************/
00112 
00113    void CCylinderEntity::EnableLEDs(CLEDMedium& c_medium) {
00114       m_pcLEDEquippedEntity->AddToMedium(c_medium);
00115    }
00116 
00117    /****************************************/
00118    /****************************************/
00119 
00120    void CCylinderEntity::DisableLEDs() {
00121       m_pcLEDEquippedEntity->RemoveFromMedium();
00122    }
00123    
00124    /****************************************/
00125    /****************************************/
00126 
00127    void CCylinderEntity::AddLED(const CVector3& c_offset,
00128                                 const CColor& c_color) {
00129       m_pcLEDEquippedEntity->AddLED(c_offset,
00130                                     GetEmbodiedEntity().GetOriginAnchor(),
00131                                     c_color);
00132       UpdateComponents();
00133    }
00134 
00135    /****************************************/
00136    /****************************************/
00137 
00138    REGISTER_ENTITY(CCylinderEntity,
00139                    "cylinder",
00140                    "Carlo Pinciroli [ilpincy@gmail.com]",
00141                    "1.0",
00142                    "A stretchable cylinder.",
00143                    "The cylinder entity can be used to model obstacles or cylinder-shaped\n"
00144                    "grippable objects. The cylinder has a red LED on the center of one\n"
00145                    "of the circular surfaces, that allows perception using the cameras.\n"
00146                    "The cylinder can be movable or not. A movable object can be pushed\n"
00147                    "and gripped. An unmovable object is pretty much like a column.\n\n"
00148                    "REQUIRED XML CONFIGURATION\n\n"
00149                    "To declare an unmovable object (i.e., a column) you need the following:\n\n"
00150                    "  <arena ...>\n"
00151                    "    ...\n"
00152                    "    <cylinder id=\"cyl1\" radius=\"0.8\" height=\"0.5\" movable=\"false\">\n"
00153                    "      <body position=\"0.4,2.3,0\" orientation=\"45,0,0\" />\n"
00154                    "    </cylinder>\n"
00155                    "    ...\n"
00156                    "  </arena>\n\n"
00157                    "To declare a movable object you need the following:\n\n"
00158                    "  <arena ...>\n"
00159                    "    ...\n"
00160                    "    <cylinder id=\"cyl1\" radius=\"0.8\" height=\"0.5\"\n"
00161                    "              movable=\"true\" mass=\"2.5\">\n"
00162                    "      <body position=\"0.4,2.3,0\" orientation=\"45,0,0\" />\n"
00163                    "    </cylinder>\n"
00164                    "    ...\n"
00165                    "  </arena>\n\n"
00166                    "The 'id' attribute is necessary and must be unique among the entities. If two\n"
00167                    "entities share the same id, initialization aborts.\n"
00168                    "The 'radius' and 'height' attributes specify the size of the cylinder. When\n"
00169                    "you add a cylinder, imagine it initially unrotated and centered in the origin.\n"
00170                    "The base of the cylinder, then, is parallel to the XY plane and its height\n"
00171                    "goes with the Z axis.\n"
00172                    "The 'movable' attribute specifies whether or not the object is movable. When\n"
00173                    "set to 'false', the object is unmovable: if another object pushes against it,\n"
00174                    "the cylinder won't move. When the attribute is set to 'true', the cylinder is\n"
00175                    "movable upon pushing or gripping. When an object is movable, the 'mass'\n"
00176                    "attribute is required.\n"
00177                    "The 'mass' attribute quantifies the mass of the cylinder in kg.\n"
00178                    "The 'body/position' attribute specifies the position of the base of the\n"
00179                    "cylinder in the arena. The three values are in the X,Y,Z order.\n"
00180                    "The 'body/orientation' attribute specifies the orientation of the cylinder. All\n"
00181                    "rotations are performed with respect to the center of mass. The order of the\n"
00182                    "angles is Z,Y,X, which means that the first number corresponds to the rotation\n"
00183                    "around the Z axis, the second around Y and the last around X. This reflects\n"
00184                    "the internal convention used in ARGoS, in which rotations are performed in\n"
00185                    "that order. Angles are expressed in degrees.\n\n"
00186                    "OPTIONAL XML CONFIGURATION\n\n"
00187                    "It is possible to add any number of colored LEDs to the cylinder. In this way,\n"
00188                    "the cylinder is visible with a robot camera. The position and color of the\n"
00189                    "LEDs is specified with the following syntax:\n\n"
00190                    "  <arena ...>\n"
00191                    "    ...\n"
00192                    "    <cylinder id=\"cyl1\" radius=\"0.8\" height=\"0.5\"\n"
00193                    "              movable=\"true\" mass=\"2.5\">\n"
00194                    "      <body position=\"0.4,2.3,0\" orientation=\"45,0,0\" />\n"
00195                    "      <leds medium=\"id_of_led_medium\">\n"
00196                    "        <led offset=\" 0.15, 0.15,0.15\" anchor=\"origin\" color=\"white\" />\n"
00197                    "        <led offset=\"-0.15, 0.15,0\"    anchor=\"origin\" color=\"red\"   />\n"
00198                    "        <led offset=\" 0.15, 0.15,0\"    anchor=\"origin\" color=\"blue\"  />\n"
00199                    "        <led offset=\" 0.15,-0.15,0\"    anchor=\"origin\" color=\"green\" />\n"
00200                    "      </leds>\n"
00201                    "    </cylinder>\n"
00202                    "    ...\n"
00203                    "  </arena>\n\n"
00204                    "In the example, four LEDs are added around the cylinder. The LEDs have\n"
00205                    "different colors and are located around the cylinder. The LEDs are\n"
00206                    "managed by the LED medium declared in the <media> section of the\n"
00207                    "configuration file with id \"id_of_led_medium\"",
00208                    "Usable"
00209       );
00210 
00211    /****************************************/
00212    /****************************************/
00213 
00214    REGISTER_STANDARD_SPACE_OPERATIONS_ON_COMPOSABLE(CCylinderEntity);
00215 
00216    /****************************************/
00217    /****************************************/
00218 
00219 }
```

---

Generated on 10 Jul 2018 for ARGoS by 
 1.6.1
